# Leukocyte presence does not increase microbicidal activity of Platelet-rich Plasma in vitro

**DOI:** 10.1186/s12866-015-0482-9

**Published:** 2015-07-30

**Authors:** Erminia Mariani, Valentina Canella, Andrea Berlingeri, Alessandra Bielli, Luca Cattini, Maria Paola Landini, Elizaveta Kon, Maurilio Marcacci, Berardo Di Matteo, Giuseppe Filardo

**Affiliations:** Laboratory of Immunorheumatology and Tissue Regeneration/RAMSES, Rizzoli Orthopaedic Institute, Via di Barbiano 1/10, 40136 Bologna, Italy; Department of Medical and Surgical Sciences, University of Bologna, Bologna, Italy; Laboratory of Biomechanics and Technology Innovation/NABI, 2nd Orthopaedic and Traumatologic Clinic, Rizzoli Orthopaedic Institute, via di Barbiano 1/10, Bologna, Italy; Unit of Clinical Microbiology, St. Orsola University Hospital, University of Bologna, Bologna, Italy

**Keywords:** Platelet-rich plasma, Leukocytes, Bacterial growth inhibition, Antimicrobial activity, Microbicidal proteins, Nosocomial infections

## Abstract

**Background:**

Human platelets are a rich reservoir of molecules that promote regenerative processes and microbicidal activity. This activity might be increased by concentration in platelet-rich plasma (PRP) products and modulated by the presence of leukocytes. Despite extensive use in clinical procedures, only few studies have investigated PRP’s real microbicidal potential. Therefore, this study aimed at comparing the *in vitro* microbicidal activity of platelets and leukocyte-enriched PRP (L-PRP) to pure platelet-rich plasma (P-PRP) and the contribution of leukocytes to microbicidal properties.

Antimicrobial effects of P- and L-PRP were tested against *Escherichia Coli*, *Staphylococcus Aureus*, *Klebsiella Pneumoniae*, *Pseudomonas Aeruginosa* and *Enterococcus Faecalis.* Furthermore, L-PRP was frozen (L-PRP cryo) to assess whether the preparation maintained *in vitro* characteristics. Microbicidal proteins released by the three preparations were also evaluated.

**Results:**

L-PRP, L-PRP cryo and P-PRP generally induced comparable bacterial growth inhibition for up to 4 h’ incubation, range 1–4 log. MIP-1α, RANTES, GRO-α, IL-8, NAP-2, SDF-1α and IL-6 showed strong microbicidal potential.

**Conclusions:**

We found *in vitro* antibacterial activity of L-PRP and P-PRP and the possibility to cryopreserve L-PRP, without important changes to its effectiveness; similar microbicidal activity between preparations containing or not leukocytes; and the contribution of three new molecules (NAP-2, SDF-1α and IL-6).

## Background

Human platelets are a rich reservoir of molecules and growth factors that can promote regenerative processes [1, 2] and stimulate tissue healing by the local release of platelet-derived growth factors and other bioactive molecules [3–6]. In addition, platelets have been associated with antibacterial host defense [7–10], due to the release of platelet microbicidal proteins (PMP) [11] and multiple chemokines that, sharing molecular structures (CC or CXC motifs) with multiple antimicrobial peptides (AMP), result in the double role of chemoattractant and microbicidal proteins [12, 13].

These proteins act as a first line of defense against invading micro-organisms and molecules released after platelet activation are also able to recruit cells (e.g. leukocytes), thus modulating *in vivo* multiple physiological processes [8].

The antibacterial potential of platelets might be increased through their concentration in platelet-rich plasma (PRP) products, thus making these blood-derivatives good candidates for preventing operative and post-operative infections, and avoiding the risk of immunological reactions because of their autologous origins. Accordingly, in recent decades various types of PRP preparations have been developed, from pure platelet-rich plasma (P-PRP, characterized by the presence of platelets only) to leukocyte- and platelet-rich plasma (L-PRP) [14, 15]. As leukocytes play an important role in the innate host-defense, the antibacterial effects of plasma enriched in platelets and leukocytes might have increased microbicidal activity compared to the fractions containing platelets alone [16].

Unfortunately, despite the extensive use of such compounds in a variety of surgical and clinical procedures [17–21], up to now only few studies have investigated platelet microbicidal activity [22–26] and, therefore, the real antibacterial potential of PRP. In addition, the many protocols for PRP preparation, the possibility to include different cellular components in various proportions and the lack of a consensus terminology (so that different acronyms identify similar preparations) make the definition of PRP biological and antibacterial properties difficult [15, 27, 28].

Accordingly, the purpose of the current study was to investigate the *in vitro* microbicidal activity of a blood-derivative enriched in platelets and leukocytes (called L-PRP) compared to pure platelet-rich plasma (P-PRP), to assess their effects and the contribution of the leukocyte component to the antibacterial properties.

The microbicidal activities of P-PRP and L-PRP were tested against *Escherichia Coli*, *Staphylococcus Aureus*, *Klebsiella Pneumoniae*, *Pseudomonas Aeruginosa* and *Enterococcus Faecalis*, as species potentially involved in bone, soft tissue and wound infections [29, 30] and representing the strains more commonly involved in nosocomial infections.

In addition, since haemoderivatives can be used for patients treatment after collection and storage, L-PRP was also frozen and then thawed to obtain the cryopreserved L-PRP (L-PRP cryo), to assess whether the preparation maintained its *in vitro* characteristics against the selected bacteria.

We showed similar *in vitro* antibacterial activity of L-PRP and P-PRP against the selected bacteria, and the possibility to cryopreserve L-PRP without important changes to its effectiveness. The contribution of three new molecules (NAP-2, SDF-1α and IL-6) as antimicrobial peptides have also been evidenced.

## Methods

### Donors

Ten healthy men (mean age ± SD: 29.9 ± 3.4 years), enrolled on a voluntary basis, signed a written informed consent form to participate in the study protocol which was approved by the Rizzoli Orthopedic Institute Ethic Committee.

Subjects with smoking habits, taking non-steroidal anti-inflammatory drugs for 5 days before blood collection, suffering from systemic disorders and/or infections, or with hemoglobin concentrations <11 g/dl and platelet numbers ≤ 150x10^3^/μl, were excluded from the study. Code numbers assigned to the samples assured subject anonymity.

### Blood-derivative preparation

P-PRP was prepared following a one-step procedure. Briefly, 45 ml of venous blood was collected from each subject in five tubes containing 1 ml of sodium citrate solution (3.8 %) as anticoagulant and centrifuged at 460 g for 8 min [31, 32], thus obtaining three layers: platelet-poor plasma (PPP) on the top of the tube, P-PRP in the middle and erythrocytes at the bottom of the tubes. In sterile conditions, about 1 ml/tube of P-PRP, located on the red blood cell pellet, was carefully harvested avoiding leukocyte collection.

L-PRP was prepared following a two-step procedure. Briefly, 150 ml of venous blood was collected from each subject in a sterile plastic bag containing 21 ml of citrate-phosphate-dextrose as anti-coagulant. After a first centrifugation step at 730 g for 15 min to separate erythrocytes, the fraction located on the red blood cell pellet was transferred in a second bag via a closed circuit and underwent a second centrifugation at 3800 g for 10 min, thus obtaining two separate layers: platelet-poor plasma (PPP), on the top of the bag and L-PRP (a concentrate of platelets and leukocytes) at the bottom. In sterile conditions, the L-PRP fraction was carefully harvested [32]. One aliquot of L-PRP was frozen at −30 °C for 2 h and then thawed thus obtaining the cryopreserved fraction (L-PRP cryo).

### Activation of platelet concentrates

Two aliquots of P-PRP, L-PRP and L-PRP cryo from each donor were activated by the addition of 10 % calcium chloride (CaCl_2,_ final concentration: 22,8 mM) and incubated for 1 h and 18 h at 37 °C in 5 % CO_2_, corresponding, respectively, to the first and last time-point of bacterial incubation. After incubation, PRP samples were centrifuged (15 min at 2800 g at 20 °C) and supernatants were collected and frozen at -30C° until use.

### Quantification of microbicidal proteins release

For the evaluation of microbicidal proteins, P-PRP, L-PRP and L-PRP-cryo from each donor were assayed in duplicate. Commercially available multiplex bead-based sandwich immunoassay kits were used to simultaneously evaluate the following soluble factors: Macrophage Inflammatory Protein (MIP)-1α (CCL3), Regulated on Activation Normal T Expressed and Secreted protein (RANTES) (CCL5), GRO-α (CXCL1), Interleukin (IL)-8 (CXCL8), Interleukin (IL)-6 (Bio-Rad Laboratories, CA, USA); neutrophil-activating protein (NAP)-2 (CXCL7), stromal cell-derived factor (SDF)-1α (CXCL12) (Milliplex MAP Kit, Millipore, Bedford, MA), as previously described [33, 34]. Briefly, distinct sets of fluorescently dyed beads loaded with capture monoclonal antibodies, specific for each cytokine to be tested, were used. Samples or standards (diluted according to the manufacturer’s recommendations) or standards (50 μl/well) were incubated with 50 μl of pre-mixed bead sets inside the wells of a 96-well microtiter plate.

The formation of different sandwich immune complexes on distinct bead sets was measured and quantified by using the Bio-Plex Protein Array System (Bio-Rad Laboratories, USA). A 50 μl volume was sampled by each well and the fluorescent signal of a minimum of 50 beads per region (chemokine/cytokine) was evaluated and recorded. Values with a coefficient of variation above the 10 % were discarded before the final data analysis.

Data were analyzed by the Bio-Plex Manager software version 6.0 (Bio-Rad Laboratories, USA). Standard levels between 70 and 130 % of the expected values were considered to be accurate and were used. In general, at least six standards were accepted and used to establish standard curves following a Five-Parameter Logistic (5-PL) regression model. Sample concentrations were immediately interpolated from the standard curves.

### Determination of platelet and leukocyte numbers

Platelet and leukocyte count was performed by the Coulter LH 750 (Beckman Coulter Inc. Miami, Fl, USA) automated hematology analyzer. Linearity was 5–1000 × 10^3^/μl for platelet count and 0.1-100 × 10^3^/μl for white blood cell count.

The median platelet number was 861x10^3^/μl (interquartile range 715-959 × 10^3^/μl) in L-PRP; 290 × 10^3^/μl (interquartile range 182–385 × 10^3^/μl) in P-PRP; 275.5 × 10^3^/μl (interquartile range 207-484 × 10^3^/μl) in L-PRP cryo. The median leukocyte count was <0.2 × 10^3^/μl in P-PRP; 5.450 × 10^3/^μl (interquartile range 5.050-6.425 × 10^3^/μl) in L-PRP; 0.550 × 10^3^/μl (interquartile range 0.425-1.275 × 10^3^/μl) in L-PRP cryo.

### Preparation of bacterial stocks

Bacterial species were obtained from the Bioresource Collection of the American Type Culture Collection (ATCC, Manassas, VA, USA), chosen according to morphological characteristics of membrane and growth [35] and as species implicated in bone and soft-tissue infections [29, 30].

Three Gram negative (*Escherichia coli,* ATCC 25922; *Pseudomonas aeruginosa* ATCC 27853; *Klebsiella pneumoniae* ATCC 700603) and two Gram positive (*Staphylococcus aureus,* ATCC 29213 and *Enterococcus faecalis,* ATCC 29212) bacterial strains were selected.

From a starting suspension of 10^8^ colony-forming units (CFU)/ml in Tryptic Soy Broth medium (TSB, MEUS S.R.L., Italy), 10-fold serial dilutions (10^6^, 10^5^ and 10^4^ CFU/ml) were prepared, thus obtaining bacterial concentrations mirroring a localized infection *in vivo* [23, 36].

### Plating assay and bacterial count

In sterile conditions, P-PRP, L-PRP and L-PRP cryo (90 μl), obtained from each donor were activated with 10 μl of CaCl_2_ (22.8 mM final concentration) and added to 900 μl of each bacterial dilution to be tested (10^4^, 10^5^, 10^6^ CFU/ml). Tubes were mixed vigorously and then incubated at 37 °C for 1, 2, 4 and 18 h respectively. TSB was used as control condition.

**Fig. 1:**
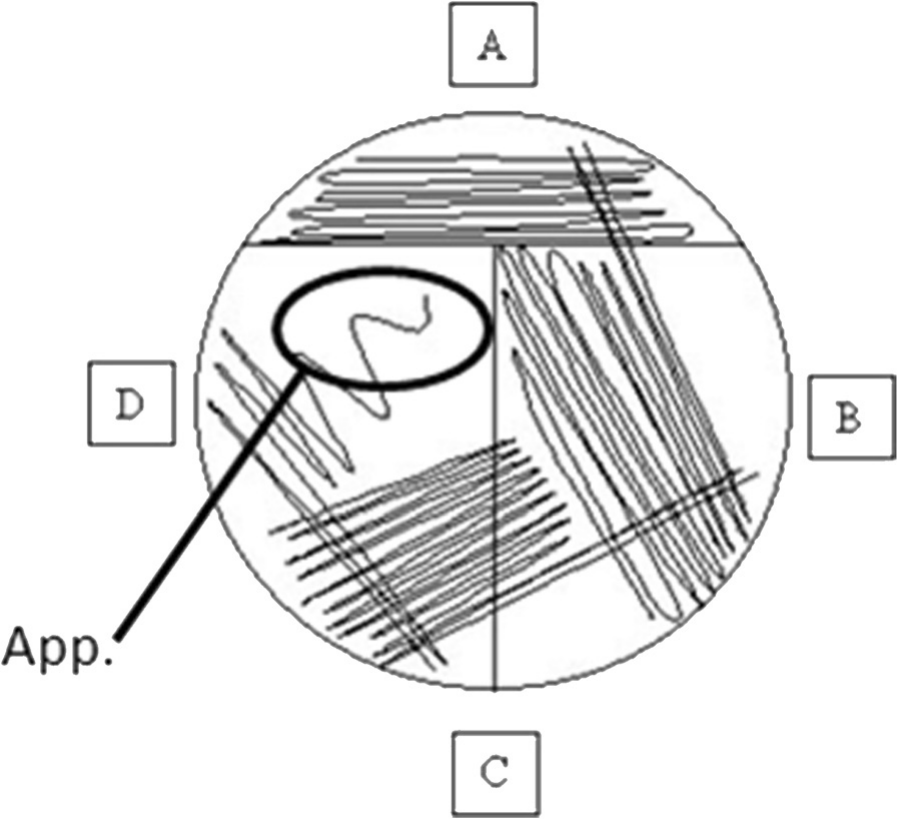
Plating pattern. **a** 1^st^ quadrant; **b** 2^nd^ quadrant; **c** 3^rd^ quadrant; **d** appendix. (Source: *OpenWetWare.org*)

After incubation, 10 μl of each bacterial suspension was spread on Blood Horse Agar (MEUS S.R.L., Italy) using a seeding distributed in 3 quadrants with an appendix in terminal part (Fig. 1). Agar plates were incubated at 37 °C for 18–24 h, then a semi quantitative estimation of bacterial growth (CFU/ml) was performed by counting the colonies in each quadrant (Table 1).

**Table 1:** Semi-quantitative estimation of bacterial growth (CFU/ml)

Number of quadrant	Colony count					
1^st^ quadrant	0	1-4	≥5	≥5	≥5	≥5
2^nd^ quadrant			<5	≥5	≥5	≥5
3^rd^ quadrant				<5	≥5	≥5
Appendix					0	≥1
Approximate growth (CFU/ml)	<10^3^	10^3^	10^4^	10^5^	10^6^	>10^6^

Bacterial growth was determined by the number of colonies detected on each agar plate

### Statistical analysis

Values are presented as medians, interquartile ranges and percentage, as appropriate.

The antimicrobial activity is calculated as difference between experimental conditions and control culture conditions and reported as log of growth inhibition. Data are expressed as percent frequency of subject displaying a certain inhibition.

Differences in growth inhibition among incubation times, types of treatment and number of seeded bacteria were analyzed using the Friedman-Anova test. Differences between two culture conditions were analyzed by the Wilcoxon-matched pair test.

Correlations between concentrations of released soluble factors and the growth inhibition of each bacterium strain at the different time points were analyzed by means of the Kendall-Tau rank correlation test.

The level of statistical significance was set at p < 0.05 and adjusted according to Bonferroni’s correction for multiple comparisons (p < 0.017), as appropriate. Data were analyzed using the Statistica 6 software (StatSoft. Inc., Tulsa, USA).

## Results

### Bacterial growth inhibition

In general, bacterial growth inhibition produced by the three PRP preparations lasted for up to 4 h of incubation, and ranged between 1 and 4 log (corresponding to inhibitions from 10 to 10.000 CFU/ml), depending on the bacterium and the experimental conditions tested. After 18 h, no growth inhibitions were observed.

**Fig. 2:**
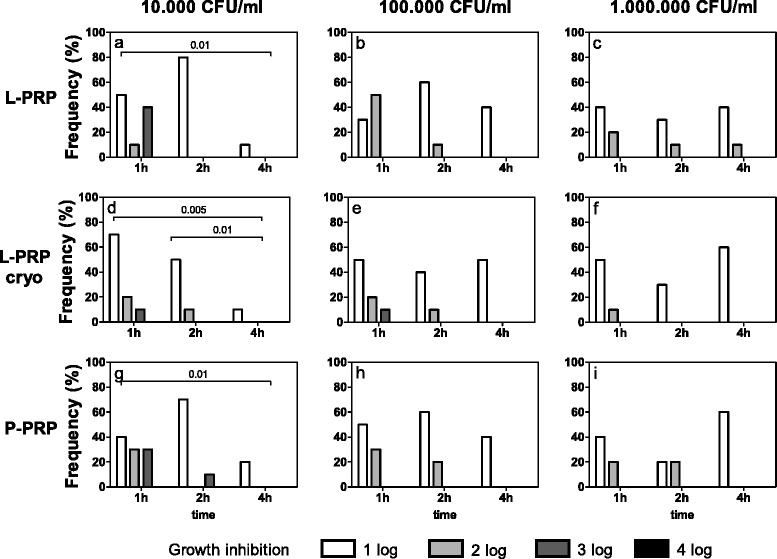
*Escherichia coli* growth inhibition. Each bacterial dilution to be tested (10^4^, 10^5^, 10^6^ CFU/ml, as reported above each panel) was incubated with L-PRP, L-PRP cryo and P-PRP at 37 °C for 1, 2, 4 h. Then each bacterial suspension was spread on Blood Horse Agar plates and incubated at 37 °C for 18–24 h. A semi quantitative estimation of bacterial growth (CFU/ml) was performed by counting the colonies in each plate quadrant (for details see Methods). The antimicrobial activity was calculated as difference between experimental conditions and control culture conditions and expressed as growth inhibition: 1log, 2log, 3log and 4log growth inhibition indicate a decrease of 10, 100, 1000 and 10000 CFU/ml respectively compared to CFU/ml indicated above each panel. Data are reported as percentage of subjects (Frequency %) displaying a certain inhibition. Growth inhibition differences among incubation times, within equal PRP preparations and bacterial concentrations. Friedman-Anova test: **a**) p < 0.0005; **b**, **e**) p < 0.05; **d**) p < 0.0002; **g**) p < 0.005; **h**) p < 0.02; **c**, **f**, **i**) not significant. Wilcoxon-matched pair test: as reported in the figure. Growth inhibition differences among bacterial concentrations, within equal incubation times and PRP preparations. Friedman-Anova test: 1 h **a**) vs **b**) vs **c**) and **g**) vs **h**) vs **i**) p < 0.01; **d**) vs **e)** vs **f**) p < 0.05; 2 h and 4 h, not significant. Wilcoxon-matched pair test: 1 h **a**) vs **c**) and **g**) vs **i**) p < 0.01. Growth inhibition differences among PRP preparations within equal bacterial concentrations and incubation times. Friedman-Anova test: not significant

*Escherichia coli* (Fig. 2a-i) showed significant growth inhibitions after treatment with L-PRP, L-PRP cryo or P-PRP for up to 4 h when 10^4^ and 10^5^ CFU/ml were seeded (Friedman-Anova test: p < 0.05, at least) (Fig. 2a, b, d, e, g, h), but not at 10^6^ CFU/ml (Fig. 2c, f, i). Whatever the PRP preparation, at 10^4^ CFU/ml a decreasing inhibiting effect was observed between 1 and 4 h of incubation (Fig. 2a, d, g) and between 2 and 4 h only with L-PRP cryo (Fig. 2d). When increasing the concentration of bacteria, the growth inhibition was similar independently of the incubation time.

Decreased growth inhibitions were also observed with increasing bacterial concentrations after 1 h on the same preparation (Friedman-Anova test: p < 0.05, at least) (Fig. 2a-c; d-f; g-i). This decrease was particularly evident between 10^4^ (Fig. 2a, c) and 10^6^ CFU/ml (Fig. 2g, i), following incubation with L- and P-PRP. No significant differences were observed among the preparations.

**Fig. 3:**
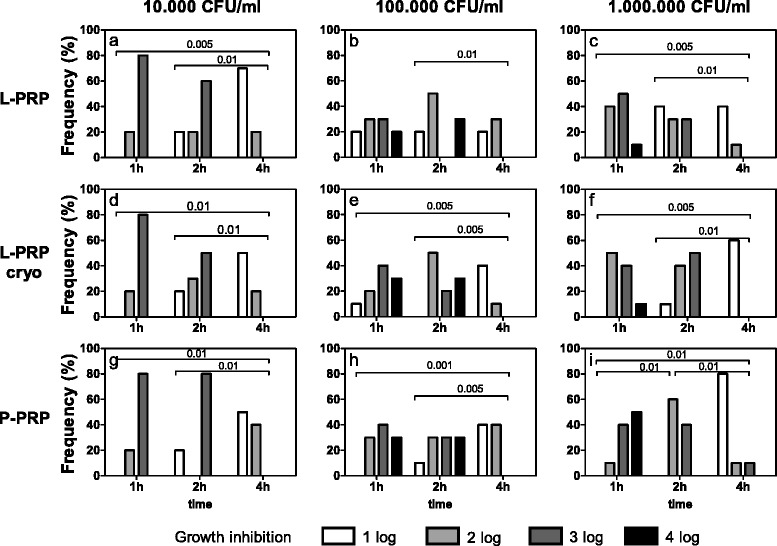
*Staphylococcus aureus* growth inhibition. Each bacterial dilution to be tested (10^4^, 10^5^, 10^6^ CFU/ml, as reported above each panel) was incubated with L-PRP, L-PRP cryo and P-PRP at 37 °C for 1, 2, 4 h. Then each bacterial suspension was spread on Blood Horse Agar plates and incubated at 37 °C for 18–24 h. A semi quantitative estimation of bacterial growth (CFU/ml) was performed by counting the colonies in each plate quadrant (for details see Methods). The antimicrobial activity was calculated as difference between experimental conditions and control culture conditions and expressed as growth inhibition: 1log, 2log, 3log and 4log growth inhibition indicate a decrease of 10, 100, 1000 and 10000 CFU/ml respectively compared to CFU/ml indicated above each panel. Data are reported as percentage of subjects (Frequency %) displaying a certain inhibition. Growth inhibition differences among incubation times, within equal PRP preparations and bacterial concentrations. Friedman-Anova test: **a**, **c**, **f**) p < 0.0002; **b**) p < 0.01; **d**, **e**, **g**) p < 0.0005; **h**) p < 0.001; **i**) p < 0.0001. Wilcoxon-matched pair test: as reported in the figure. Growth inhibition differences among bacterial concentrations, within equal incubation times and PRP preparations. Friedman-Anova test: not significant. Growth inhibition differences among PRP preparations within equal bacterial concentrations and incubation times. Friedman-Anova test: **c**) vs **f**) vs **i**) 1 h p < 0.002; 2 h p < 0.02; 4 h p < 0.01. Wilcoxon-matched pair test: not significant

*Staphylococcus aureus* (Fig. 3) was significantly inhibited for at least up to 4 h, independently of the preparation tested or the number of seeded bacteria (Friedman-Anova test: p < 0.01, at least) (Fig. 3a-i). For almost all the experimental conditions, the inhibition of growth was similar during the first two hours of incubation (Fig. 3a-h), whereas it decreased between 1 and 4 h (Fig. 3a, c-i) and between 2 and 4 h (Fig. 3a-i), excluding 10^5^ CFU/ml bacteria treated with L-PRP (Fig. 3b). Independently of the preparation, increasing concentrations of bacteria underwent similar inhibitions of growth when incubated for the same time.

L-PRP, L-PRP cryo and P-PRP induced similar growth inhibitions at 10^4^ and 10^5^ CFU/ml (Fig. 3a, d, g and b, e, h) for each incubation time, whereas significant differences were observed at 10^6^ CFU/ml (Friedman-Anova test: p < 0.02, at least) (Fig. 3c, f, i).

**Fig. 4:**
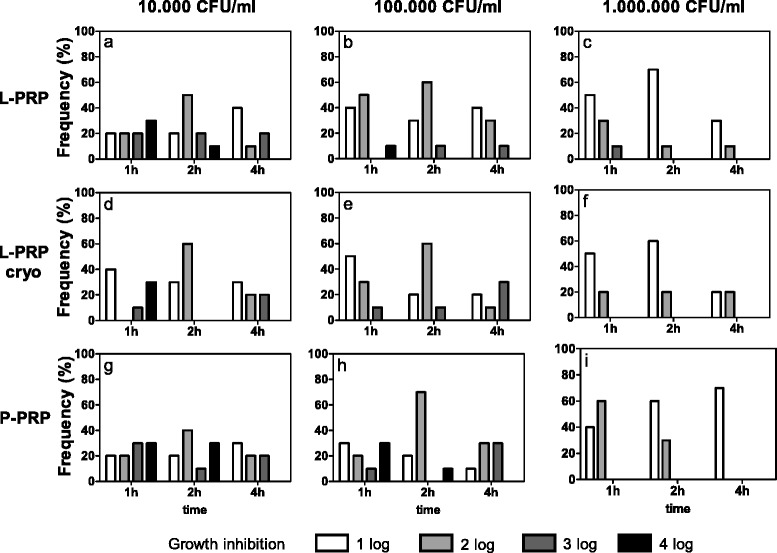
*Klebsiella pneumoniae* growth inhibition. Each bacterial dilution to be tested (10^4^, 10^5^, 10^6^ CFU/ml, as reported above each panel) was incubated with L-PRP, L-PRP cryo and P-PRP at 37 °C for 1, 2, 4 h. Then each bacterial suspension was spread on Blood Horse Agar plates and incubated at 37 °C for 18–24 h. A semi quantitative estimation of bacterial growth (CFU/ml) was performed by counting the colonies in each plate quadrant (for details see Methods). The antimicrobial activity was calculated as difference between experimental conditions and control culture conditions and expressed as growth inhibition: 1log, 2log, 3log and 4log growth inhibition indicate a decrease of 10, 100, 1000 and 10000 CFU/ml respectively compared to CFU/ml indicated above each panel. Data are reported as percentage of subjects (Frequency %) displaying a certain inhibition. Growth inhibition differences among incubation times, within equal PRP preparations and bacterial concentrations. Friedman-Anova test: **a**-**h**) not significant; **i**) p < 0.02. Wilcoxon-matched pair test: not significant. Growth inhibition differences among bacterial concentrations, within equal incubation times and PRP preparations. Friedman-Anova test: 1 h **a**) vs **b**) vs **c**) and **g**) vs **h**) vs **i**) p < 0.05; **d**) vs **e**) vs **f**) not significant; 2 h **a**) vs **b**) vs **c**) and **g**) vs **h**) vs **i**) p < 0.01; **d**) vs **e**) vs **f**) p < 0.05; 4 h **a**) vs **b**) vs **c**) p < 0.01; **d**) vs **e**) vs **f**) p < 0.05; **g**) vs **h**) vs **i**) not significant. Wilcoxon-matched pair test: 2 h *g)* vs **i**) p < 0.01. Growth inhibition differences among PRP preparations within equal bacterial concentrations and incubation times. Friedman-Anova test: **a**) vs **d**) vs **g**) 1 h p < 0.02; 2 h p < 0.05; **b**) vs **e**) vs **h**) 1 h p < 0.05; **c**) vs **f**) vs **i**) 1 h p < 0.05. Wilcoxon-matched pair test: not significant

*Klebsiella pneumoniae* did not show significant time-dependent growth inhibition within each preparation for up to 4 h whatever the concentration of seeded bacteria (Fig. 4a-h), excluded P-PRP at 10^6^ CFU/ml (Friedman-Anova test: p < 0.02) (Fig. 4i). Within each preparation, different growth inhibitions were observed among the three bacterial concentrations after 1, 2 and 4 h with L-PRP (Fig. 4a-c), after 2 and 4 h with L-PRP cryo (Fig. 4d-f) and after 1 and 2 h with P-PRP (Friedman-Anova test: p < 0.05, at least) (Fig. 4g-i). Significant differences were also observed comparing the preparations after 1 and 2 h when seeding 10^4^ CFU/ml (Fig. 4a, d, g), but only after 1 h at higher bacterial concentrations (Fig. 4b, e, h and c, f, i) (Friedman-Anova test: p < 0.05).

**Fig. 5:**
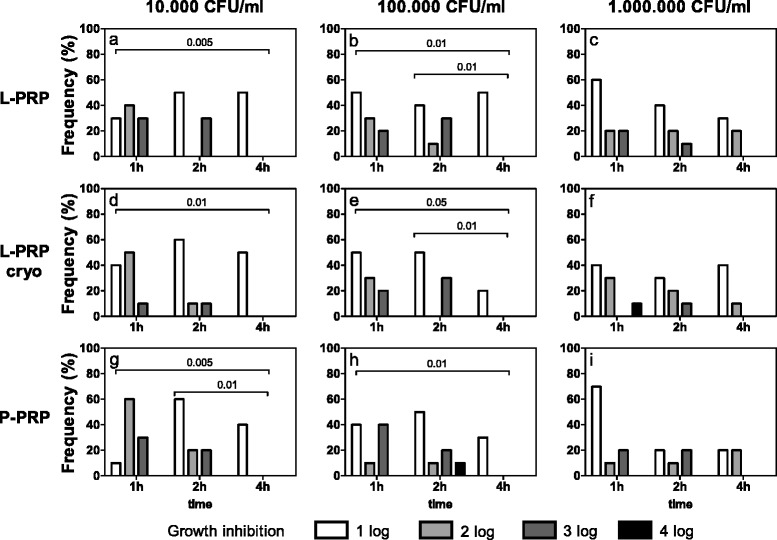
*Pseudomonas aeruginosa* growth inhibition. Each bacterial dilution to be tested (10^4^, 10^5^, 10^6^ CFU/ml, as reported above each panel) was incubated with L-PRP, L-PRP cryo and P-PRP at 37 °C for 1, 2, 4 h. Then each bacterial suspension was spread on Blood Horse Agar plates and incubated at 37 °C for 18–24 h. A semi quantitative estimation of bacterial growth (CFU/ml) was performed by counting the colonies in each plate quadrant (for details see Methods). The antimicrobial activity was calculated as difference between experimental conditions and control culture conditions and expressed as growth inhibition: 1log, 2log, 3log and 4log growth inhibition indicate a decrease of 10, 100, 1000 and 10000 CFU/ml respectively compared to CFU/ml indicated above each panel. Data are reported as percentage of subjects (Frequency %) displaying a certain inhibition. Growth inhibition differences among incubation times, within equal PRP preparations and bacterial concentrations. Friedman-Anova test: **a**) p < 0.01; **b**, **e**) p < 0.001; **d**, **h**) p < 0.002; **g**) p < 0.0005; **c**, **f**, **i**) not significant. Wilcoxon-matched pair test: as reported in the figure. Growth inhibition differences among bacterial concentrations, within equal incubation times and PRP preparations. Friedman-Anova test: 1 h and 4 h for all the PRP preparations, not significant; 2 h **g**) vs **h**) vs **i**) p < 0.05; **a**) vs **b**) vs **c**) and **d**) vs **e**) vs **f**) not significant. Wilcoxon-matched pair test: not significant. Growth inhibition differences among PRP preparations within equal bacterial concentrations and incubation times. Friedman-Anova test: not significant

*Pseudomonas aeruginosa* showed significant growth inhibition with time, when 10^4^ (Fig. 5a, d, g) and 10^5^ CFU/ml (Fig. 5b, e, h) were seeded (Friedman-Anova test: p < 0.01, at least), independently of the preparations. Growth inhibition differences were observed between 1 (Fig. 5a, b, d) and 4 h of incubation (Fig. 5e, g, h) and between 2 and 4 h (Fig. 5b, e, g). No growth differences were found when 10^6^ CFU/ml were seeded (Fig. 5c, f, i). After incubation with L-PRP and L-PRP cryo, similar growth inhibitions were observed among the bacterial concentrations on the same time, whereas a significant decrease was found after 2 h of incubation with P-PRP (Friedman-Anova test: p < 0.05) (Fig. 5g-i). No significant differences of growth inhibition were observed among the preparations.

**Fig. 6:**
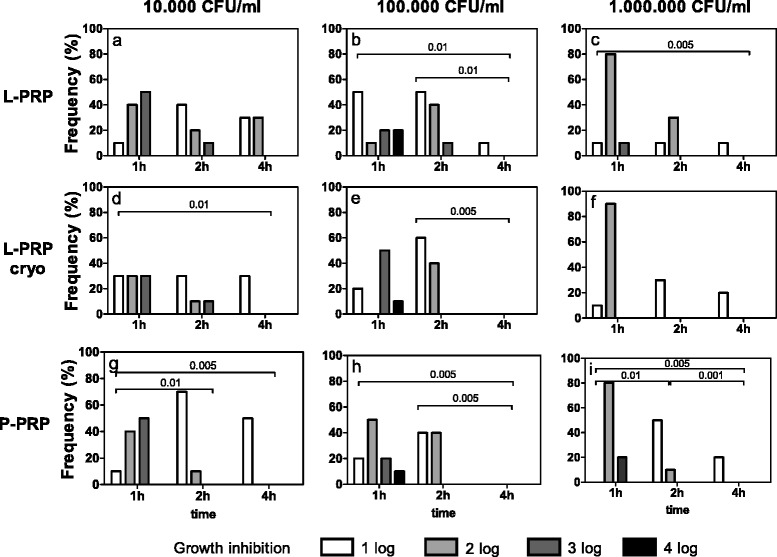
*Enterococcus faecalis* growth inhibition. Each bacterial dilution to be tested (10^4^, 10^5^, 10^6^ CFU/ml, as reported above each panel) was incubated with L-PRP, L-PRP cryo and P-PRP at 37 °C for 1, 2, 4 h. Then each bacterial suspension was spread on Blood Horse Agar plates and incubated at 37 °C for 18–24 h. A semi quantitative estimation of bacterial growth (CFU/ml) was performed by counting the colonies in each plate quadrant (for details see Methods). The antimicrobial activity was calculated as difference between experimental conditions and control culture conditions and expressed as growth inhibition: 1log, 2log, 3log and 4log growth inhibition indicate a decrease of 10, 100, 1000 and 10000 CFU/ml respectively compared to CFU/ml indicated above each panel. Data are reported as percentage of subjects (Frequency %) displaying a certain inhibition. Growth inhibition differences among incubation times, within equal PRP preparations and bacterial concentrations. Friedman-Anova test: **a**, **d**) p < 0.01; **b**, **c**) p < 0.001; **e**) p < 0.002; **f**, **g**) p < 0.0005; **h**, **i**) p < 0.0001. Wilcoxon-matched pair test: as reported in the figure. Growth inhibition differences among bacterial concentrations, within equal incubation times and PRP preparations. Friedman-Anova test: 2 h **a**) vs **b**) vs **c**) p < 0.005; **d**) vs **e**) vs **f**) p < 0.001; **g**) vs **h**) vs **i**) not significant; 4 h **a**) vs **b**) vs **c**) p < 0.05; **d**) vs **e**) vs **f**) p < 0.02; **g**) vs **h**) vs **i**) p < 0.05; 1 h not significant. Wilcoxon-matched pair test: 2 h **e**) vs **f**) p < 0.005. Growth inhibition differences among PRP preparations within equal bacterial concentrations and incubation times. Friedman-Anova test: **b**) vs **e**) vs **h**) 2 h p < 0.05. Wilcoxon-matched pair test: not significant

*Enterococcus faecalis* growth was inhibited by all the preparations for up to 4 h of incubation, whatever the bacterial concentrations tested (Friedman-Anova test: p < 0.01, at least) (Fig. 6a-i). Differences were observed between 1 and 4 h when 10^4^ CFU/ml were seeded with L-PRP cryo and P-PRP (Fig. 6d, g); at 10^5^ CFU/ml with L-PRP and P-PRP (Fig. 6b, h) and at 10^6^ CFU/ml with all the preparations (Fig. 6c, f, i). Moreover, differences of growth inhibition were observed between 1 and 2 h when 10^4^ CFU/ml were seeded with P-PRP (Fig. 6g) and when 10^6^ CFU/ml were incubated with L-PRP cryo and P-PRP (Fig. 6f, i). Between 2 and 4 h, inhibition was evident at 10^5^ CFU/ml incubated with L-PRP and P-PRP (Wilcoxon-matched pair test: p < 0.01, at least) (Fig. 6b, h) and at 10^6^ CFU/ml incubated with P-PRP (Wilcoxon-matched pair test: p < 0.001) (Fig. 6i). Independently of the preparation, significant variations among the different bacterial concentrations were found after 2 and 4 h of incubation with L-PRP and L-PRP cryo and only after 4 h with P-PRP (Friedman-Anova test: p < 0.05, at least) (Fig. 6a-c, d-f and g-i), with significant differences between 10^5^ and 10^6^ CFU/ml after 2 h of incubation with L-PRP cryo (Fig. 6*e, f*). The comparison among the three plasma fractions evidenced a decreasing inhibiting activity (from L-PRP to P-PRP) following the incubation of 10^5^ CFU/ml at 2 h (Friedman-Anova test: p < 0.05) (Fig. 6b, e, h).

### Microbicidal protein evaluation

**Fig. 7:**
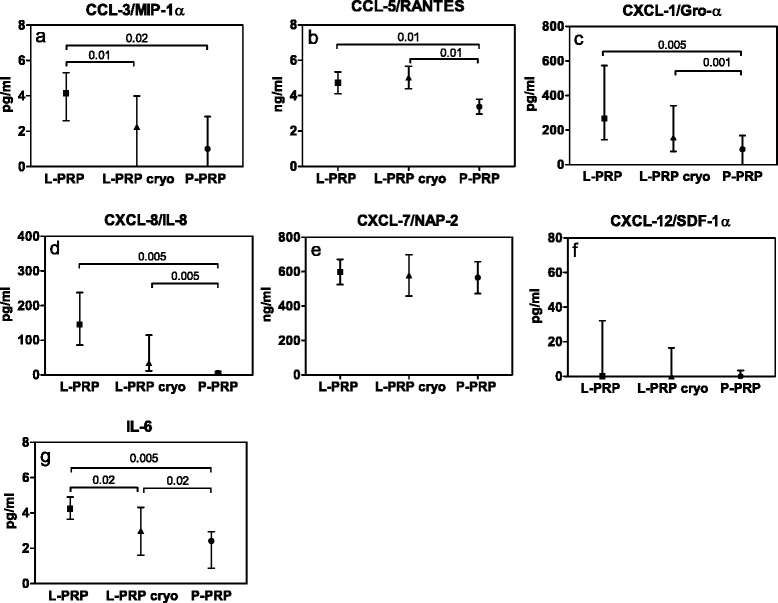
Microbicidal protein concentrations in L-PRP, L-PRP cryo and P-PRP after 1 h of incubation. Results are expressed as medians and interquartile ranges. Friedman-Anova test: **a**, **b**) p < 0.01; **c**, **d**) p < 0.0005; **g**) p < 0.001; **e**, **f**) n.s. Wilcoxon-matched pair test: as reported in the figure

The concentrations of the main soluble factors, described as microbicidal proteins, were different among the three PRP preparations (as determined by Friedman-Anova test: p < 0.02, at least) (Fig. 7a-d, g), excluded NAP-2 and SDF-1α (Fig. 7e, f), whose concentrations were similar.

In general, the molecules were more concentrated in L-PRP (MIP-1α, RANTES, GRO-α, IL-8 and IL-6) and in L-PRP cryo (RANTES, GRO-α, IL-8 and IL-6) than in P-PRP (Wilcoxon-matched pair test: p < 0.02 at least).

The concentrations of RANTES, GRO-α and IL-8 were stable after cryopreservation, whereas they were slightly decreased in MIP-1α and IL-6 (Wilcoxon-matched pair test: p < 0.02), as determined by the comparison between L-PRP and L-PRP cryo.

After 18 h of activation, IL-8, RANTES and GRO-α concentrations increased in all the preparations, whereas NAP-2 and SDF-1α were stable (data not shown). IL-6 concentration increased only in L-PRP and L-PRP cryo, whereas MIP-1α increased in L-PRP and P-PRP, but not L-PRP cryo (data not shown).

For all the bacteria, we evaluated the correlation between bacterial growth inhibition (induced by the three PRP preparations obtained by each donor after 1, 2 and 4 h of incubation) and the concentration of microbicidal proteins released by the matched PRP preparations after 1 h of activation (Table 2). It was not possible to analyze the corresponding correlations after 18 h, since the bacteria reached an overgrowth phase.

**Table 2:** Correlations between bacterial growth inhibition and soluble factors

Bacteria	Time	Soluble factors						
		CCL3/MIP-1α	CCL-5/RANTES	CXCL1/GRO-α	CXCL-8/IL-8	CXCL-7/NAP2	CXCL-12/SDF-1α	IL-6
Escherichia coli	1 h	---	---	---	---	---	p < 0.05	---
	2 h	---	p < 0.05	p < 0.05	---	---	p < 0.005	---
	4 h	---	---	p < 0.01	---	---	---	---
Staphylococcus aureus	1 h	p < 0.01	---	p < 0.0001	---	p < 0.01	---	---
	2 h	p < 0.005	p < 0.01	---	p < 0.01	p < 0.000005	p < 0.05	---
	4 h	p < 0.005	p < 0.05	---	p < 0.01	p < 0.00001	---	---
Klebsiella pneumoniae	1 h	---	p < 0.000005	p < 0.01	p < 0.01	p < 0.001	p < 0.0001	p < 0.01
	2 h	p < 0.01	---	---	---	---	---	---
	4 h	p < 0.01	p < 0.05	---	p < 0.005	p < 0.000005	p < 0.001	p < 0.005
Pseudomonas aeruginosa	1 h	---	---	---	---	---	---	---
	2 h	p < 0.000005	p < 0.0005		p < 0.01	p < 0.000005	p < 0.005	p < 0.0001
	4 h	p < 0.0005	p < 0.0005	p < 0.05	p < 0.005	p < 0.000005	p < 0.001	p < 0.0005
Enterococcus faecalis	1 h	---	---	---	---	p < 0.005	---	---
	2 h	p < 0.000005	p < 0.00005	p < 0.05	p < 0.001	p < 0.0005	p < 0.00005	p < 0.000005
	4 h	p < 0.05	---	---	---	---	---	---

Significant correlations between bacterial growth inhibition at different time points and the concentration of soluble factors released by the PRP preparations after 1 h of activation and described with antimicrobial potential, as determined by Kendall-Tau rank correlation test

The concentrations of the molecules considered (MIP-1α/CCL3, RANTES/CCL5, GRO-α/CXCL1, NAP-2/CXCL7, IL-8/CXCL8, SDF-1α/CXCL12 and IL-6) were strongly correlated to bacterial growth inhibition, mainly from the second hour of incubation (Table 2).

*Escherichia coli* inhibition showed correlations with RANTES, GRO-α and SDF-1α concentrations (Kendall-Tau rank correlation: p < 0.05, at least). *Staphylococcus aureus* inhibition correlated with the concentrations of all the molecules excluded IL-6 (Kendall-Tau rank correlation: p < 0.05, at least), whereas *Klebsiella pneumoniae*, *Pseudomonas aeruginosa* and *Enterococcus faecalis* inhibition correlated with the concentrations of all the microbicidal molecules considered (Kendall-Tau rank correlation: p < 0.05, at least).

## Discussion

In this study, the antibacterial activity of L-PRP (leukocyte and platelet-rich plasma) and P-PRP (pure platelet-rich plasma) was evaluated against five bacteria strains (*Escherichia coli, Staphylococcus aureus, Klebsiella pneumoniae, Pseudomonas aeruginosa, Enterococcus faecalis*). The contribution of leukocytes and the preservation of the biological properties after freezing were also evaluated.

We found a time-dependent inhibition of bacteria growth, for up to 4 h, at low bacterial numbers in *Escherichia coli* and *Pseudomonas aeruginosa* and at higher numbers in *Staphylococcus aureus* and *Enterococcus faecalis*, after treatment with the three plasma fractions (L-PRP, L-PRP cryo or P-PRP). *Klebsiella pneumoniae* was the only strain not showing a time-dependent inhibition of growth whatever bacterial concentration and PRP preparation.

The loss of antibacterial effect displayed by all the preparations after 18 h suggests a short-term and not complete microbicidal activity at the experimental concentrations tested, and/or that the amount of microbicidal proteins present in PRP preparation is not sufficient to limit the bacteria growth for a long time, which gives important indications for the early prophylactic use of PRP in the clinical practice.

Previous data have shown that analogous PRP formulations, enriched in leukocytes, had similar antimicrobial effects against *Escherichia coli* [22, 25]*, Staphylococcus aureus* [22–24] and *Pseudomonas aeruginosa* [25], whereas the data concerning *Klebsiella pneumoniae* are not univocal, thus indicating both partial [25] and lack of susceptibility [22].

The analysis of the distribution of growth inhibitions (as indicated by log values) identified *Staphylococcus aureus* and *Klebsiella pneumoniae* as the most susceptible bacteria, which underwent a decrease of 10.000 CFU/ml (4 logarithms of growth inhibition) in various experimental conditions. Since these two strains are well-known for their strong resistance to antibiotics [37, 38], the present results are particularly relevant for the early prophylaxis against possible bacterial contaminations during clinical applications of PRP.

The similar microbicidal activity displayed by both L-PRP and L-PRP cryo, against all the strains considered, demonstrated the maintenance of the antimicrobial properties of L-PRP even after freezing and opens the possibility for cryopreserving this preparation. PRP containing only platelets was found to maintain coagulation ability, platelet morphology, P-selectin expression and growth factor release after some hours at room temperature [39]. The cryopreservability for up to 3 months of the main proteins involved in ocular surface healing [40] and their potential use as an alternative to fetal bovine serum for the cryo-preservation of human mesenchymal stem cells [41] were also reported. Furthermore, it was recently described that the freeze-thawing storage procedure did not affect the anabolic effects on different cell types [17].

To our knowledge, this is the first description of the preservation of microbicidal properties following freezing and these data are of particular interest, considering the possibility of a single preparation of self-derived platelet products to be used in consecutive administrations for therapeutic purposes.

The inclusion and the possible role played by leukocytes in different PRP preparations have been variously debated during the last decades. Previous studies have shown mixed results, some reporting that concentrated delivery of leukocytes to a site of injury may amplify the release of anabolic and pro-inflammatory mediators [42], whereas others sustaining that leukocytes enhance the release of growth factors and anti-inflammatory mediators, thus possibly playing an important antibacterial role. Indeed, although leukocytes have been proposed as an additional source for cytokines, microbicidal proteins and myeloperoxidase activity [43], nowadays the few available published data do not support the increased microbicidal activity of PRP due to the leukocyte component [22–24]. In particular, no correlation between microbicidal activity and the number of either platelets or leukocytes in PRP preparations used against the same strains we tested and between myeloperoxidase activity and bacterial killing of PRP preparations against *Staphylococcus aureus* were observed [22, 23]. Furthermore, two PRP products, containing or lacking the leukocyte component, showed similar antimicrobial activities [24]. In agreement, we found that the bacterial growth inhibition was similar among the three plasma fractions, thus strongly suggesting that the presence of leukocytes does not supply a substantial improvement in the antibacterial potential in PRP, with the main effects probably due to platelet microbicidal molecules.

Considering this aspect, we evaluated the correlation between bacterial growth inhibition and the concentration of some soluble factors displaying microbicidal activity [12, 13]. Among these, MIP-1α, RANTES, GRO-α, IL-8, NAP-2 and SDF-1α are the most common, with molecular structures of classical chemokines and therefore called “kinocidins”, for their dual role as chemokines and microbicidal effectors [8, 13].

We obtained interesting results concerning further correlations among *Escherichia coli*, *Klebsiella pneumoniae* and *Pseudomonas aeruginosa* growth inhibition and MIP-1α, RANTES, GRO-α and IL-8 concentrations compared to previous preliminary data on a pure-PRP preparation [44]. Moreover, a new molecule (SDF-1α), previously not evaluated [44], showed correlations with *Escherichia coli* growth inhibition. In different *in vitro* experiments, strong antimicrobial effects against *Escherichia coli* were found for GRO-α but not for RANTES, although the authors used recombinant peptides at micromolar concentrations [12]. Native chemokine concentrations in serum are basically in the picomolar to nanomolar range [45], as we detected in plasma concentrates, therefore a lack of concordance with results proposed by Yang [12] might be attributable to the use of synthetic proteins instead of native molecules. In addition, since we detected significant differences of RANTES concentrations among the three PRP preparations, the lack of correlation observed with *Escherichia coli* inhibition [44] suggests the need for a threshold level for RANTES to be effective as microbicidal protein. Moreover, as this chemokine possesses chemotactic activity *in vivo*, we can speculate a microbicidal loop in which the presence of RANTES in injected PRP directly inhibits *Escherichia coli* growth and simultaneously amplifies the response by attracting and recruiting leukocytes into the sites of inflammation.

MIP-1α, RANTES and GRO-α concentrations were confirmed to correlate with growth inhibition of S*taphylococcus aureus, Pseudomonas aeruginosa* and *Enterococcus faecalis* [44]. In addition, we found that MIP-1α, RANTES, GRO-α and IL-8 were involved in the inhibition of *Klebsiella pneumoniae*, whereas IL-8 was involved in the inhibition of *Pseudomonas aeruginosa*. All these chemokines showed concentrations increasing from P-PRP to L-PRP, therefore suggesting a concentration-dependent effect of these factors on bacterial growth inhibition.

Three new molecules (NAP-2, SDF-1α and IL-6) considered displayed strong correlations with growth inhibition of almost all the bacterial analyzed.

The neutrophil-activating peptide (NAP)-2 is a derivative of platelet basic protein (PBP), which is itself part of the platelet microbicidal proteins (PMPs). NAP-2 is also well-known as activator of neutrophils (activity shared with the homologous IL-8 and GRO-α chemokines). After platelet activation, NAP-2 is released and processed at the N-terminal part, finally reaching the active form as neutrophil activating peptide (NAP-2) [46]. During infections, proteolytic activation of NAP-2 and neutrophil attraction lead to an amplified response, resulting in bacterial killing [47].

SDF (stromal cell-derived growth factor)-1α is a small chemokine, usually induced after stimulus with lipopolysaccharide (a large molecule contained in the Gram negative cell wall bacteria) [48]. SDF-1α is involved in chemotaxis and is also considered to be the main chemo attractant factor for the stem cells [49]. Its modulation was proposed to be a useful therapeutic strategy for the stimulation of tissue repair. The present results, showing correlations between SDF-1α concentrations and the growth inhibition of all the bacteria tested, open the possibility to consider this molecule as an important component within PRP preparations, thus displaying a double role both as prophylactic molecule against invading microorganisms and regulator of stem cell mobilization and therefore tissue remodeling, considering the clinical use of PRP.

IL-6 is a cytokine, secreted by macrophages and T cells [50], thus displaying an important role in host defense and possibly during infections associated to antibiotic resistant microorganisms, such as *Klebsiella pneumoniae* and *Enterococcus faecalis*. In fact, it has been demonstrated that IL-6 is involved in resistance to *Streptococcus pneumoniae* infection in a mouse model and the knockout of the IL-6 gene, inducing an increase in the levels of pro-inflammatory (TNFα, IL-1β, IFN-γ) and ant-inflammatory (IL-10) cytokines [51], resulted in the mouse’s premature death. Although the mechanisms by which IL-6 contributes to antibacterial activity are still not clear, the role of IL-6 against infection is commonly acknowledged [52].

Results presented in the present study, showing a strong correlation between IL-6 concentrations and *Klebsiella pneumoniae*, *Pseudomonas aeruginosa* and *Enterococcus faecalis* growth inhibition, support the role of IL-6 against bacterial infections.

## Conclusions

In conclusion, we have shown the *in vitro* antibacterial activity of L-PRP and P-PRP against five different bacterial strains and the possibility to cryopreserve L-PRP, without important changes in its effectiveness, thus allowing a single preparation to be used for multiple *in vivo* administrations. Furthermore, we did not find significant differences in microbicidal activity between preparations containing leukocytes before and after storage conditions, thus highlighting potential clinical uses for therapeutic protocols, including soft tissue and orthopedic surgery, or for the prevention of local infections.

The prevalent role of MIP-1α, RANTES, GRO-α and IL-8 as mediators of bacterial inhibition was confirmed. In addition, the microbicidal contribution *in vitro* of three new molecules (NAP-2, SDF-1α and IL-6) was highlighted.

Results in the present study supply basic information about the most common molecules recognized as microbicidal effectors and released by platelets and leukocytes during immunological responses, however there are obvious limitations compared to *in vivo* conditions, where a complexity of physiological networks and molecular mechanisms take place. Indeed, the initial *in loco* antimicrobial activity of some platelet-stored and leukocyte-derived chemokines contained in PRP, may be supported by the chemotactic recruitment of specific cellular populations and their interaction might amplify different prophylactic responses against infection agents and trigger specific immunological pathways.

## Abbreviations

AMP: Anti-Microbial Peptides
CaCl_2_: Calcium Chloride
CFU: Colony-Forming Units
CXCL: Chemokine (C-X-C motif) Ligand
GRO-α: Growth-regulated protein homolog alpha
IL-6: Interleukin-6
IL-8: Interleukin-8
MIP-1α: Macrophage Inflammatory Protein-1-alpha
NAP-2: Neutrophil-Activating Protein-2
PMP: Platelet Microbicidal Proteins
PPP: Platelet-Poor Plasma
PRP: Platelet-Rich Plasma
P-PRP: Pure Platelet-Rich Plasma
L-PRP: Leukocytes- and platelet-rich plasma
RANTES: Regulated on activation, normal T cell expressed and secreted
SDF-1α: Stromal cell-derived factor 1-alpha
